# The Effect of Black Tea on Blood Pressure: A Systematic Review with Meta-Analysis of Randomized Controlled Trials

**DOI:** 10.1371/journal.pone.0103247

**Published:** 2014-07-31

**Authors:** Arno Greyling, Rouyanne T. Ras, Peter L. Zock, Mario Lorenz, Maria T. Hopman, Dick H. J. Thijssen, Richard Draijer

**Affiliations:** 1 Unilever Research & Development Vlaardingen, Vlaardingen, The Netherlands; 2 Department of Physiology, Radboud University Nijmegen Medical Centre, Nijmegen, The Netherlands; 3 Top Institute Food and Nutrition, Wageningen, The Netherlands; 4 Department of Cardiology and Angiology, Campus Mitte, Charité - Universitätsmedizin, Berlin, Germany; 5 DZHK (German Centre for Cardiovascular Research), Berlin, Germany; 6 Research Institute for Sport and Exercise Sciences, Liverpool John Moores University, Liverpool, United Kingdom; University of Perugia, Italy

## Abstract

**Objective:**

Epidemiological evidence has linked consumption of black tea, produced from *Camellia sinensis*, with a reduced risk of cardiovascular diseases. However, intervention studies on the effects of tea consumption on blood pressure (BP) have reported inconsistent results. Our objective was to conduct a systematic literature review with meta-analysis of controlled human intervention studies examining the effect of tea consumption on BP.

**Methods:**

We systematically searched Medline, Biosis, Chemical Abstracts and EMBASE databases through July 2013. For inclusion, studies had to meet the following pre-defined criteria: 1) placebo controlled design in human adults, 2) minimum of 1 week black tea consumption as the sole intervention, 3) reported effects on systolic BP (SBP) or diastolic BP (DBP) or both. A random effects model was used to calculate the pooled overall effect of black tea on BP.

**Results:**

Eleven studies (12 intervention arms, 378 subjects, dose of 4–5 cups of tea) met our inclusion criteria. The pooled mean effect of regular tea ingestion was −1.8 mmHg (95% CI: −2.8, −0.7; P = 0.0013) for SBP and −1.3 mmHg (95% CI: −1.8, −0.8; P<0.0001) for DBP. In covariate analyses, we found that the method of tea preparation (tea extract powders *versus* leaf tea), baseline SBP and DBP, and the quality score of the study affected the effect size of the tea intervention (all P<0.05). No evidence of publication bias could be detected.

**Conclusions:**

Our meta-analysis indicates that regular consumption of black tea can reduce BP. Although the effect is small, such effects could be important for cardiovascular health at population level.

## Introduction

Blood pressure is acknowledged to bear an independent continuous relationship with the occurrence of cardiovascular events [1]. Furthermore hypertension is recognised as the leading global risk factor for mortality [2]. Considering this, even relatively small reductions in population blood pressure can be considered of major interest to public health [2]–[4].

A high dietary flavonoid intake has been linked to a reduced risk of CVD [5], possibly through effects on BP and endothelial function [6], [7]. Black tea, produced by fermentation of the leaves of *Camellia sinensis*, is the major source of dietary flavonoids in many countries [8]–[10].

Epidemiological data support a link between black tea consumption and a reduced risk of CVD [11]. Possibly, a BP lowering effect may contribute to the reduced risk of CVD associated with regular tea consumption [12], although results from intervention studies examining effects of tea on BP are inconsistent [13]–[19]. Indeed, previous meta-analyses have found no significant effect of black tea consumption on BP [16], [19]. Several high quality randomized controlled trials have however been published since [13]–[15], [17], [18]. Our objective was therefore to perform an up-to-date systematic review with meta-analysis of controlled human intervention studies investigating the effects of black tea consumption on BP.

## Methods

This systematic review and meta-analysis was conducted in accordance with the Preferred Reporting Items for Systematic Reviews and Meta-Analyses (PRISMA) statement guidelines. The associated checklist (Checklist S1) can be found in the supporting information section.

### Search strategy

The Medline, Biosis, Chemical Abstracts and EMBASE databases were searched (from the date of inception until July 2013) to identify potentially relevant studies conducted in human adults and published in the English language. Titles, abstracts and keywords were searched for the following terms: *Tea, black tea, flavonoid(s), tea extract(s), tea component(s), tea solid(s), camellia sinensis, blood pressure, hypertension, hypertensive, hypotension, hypotensive, endothelium, endothelial function, endothelial dysfunction*. Bibliographies from obtained publications were searched for additional potentially relevant studies.

### Selection of trials

To qualify for inclusion, studies had to meet the following pre-defined inclusion criteria: 1) randomized controlled trial in human adults; 2) use of freshly brewed black tea or black tea powder produced by drying freshly brewed tea (not purified or isolated substances from tea) for ≥ 1 week; 3) reported effects on SBP or DBP or both, along with measures of variability; 4) no intentional co-intervention from which the effect of tea could not be isolated.

We followed a two-step process for selection of studies to be included in the meta-analysis. During the first step, titles, abstracts and keywords of publications were screened to identify potentially eligible studies. During the second step, the full texts of these publications were examined to gauge eligibility based on the abovementioned inclusion criteria. Eligibility was assessed separately by two authors (AG and RD). In case of discrepancy, eligibility was discussed among authors until consensus was reached.

### Data extraction

After eligible publications were identified, the following information was extracted: 1) publication details (author, year of publication, country); 2) study design characteristics (crossover or parallel, blinding, duration); 3) subject characteristics (age, BP medication use, gender and health status); 4) treatment characteristics (type and dosage of tea, type of control); 5) BP measurement characteristics (office or ambulatory BP); 6) SBP and DBP data (including measures of variance), and 7) characteristics needed to gauge study quality. If any of the required data were not reported, the authors of the publication in question were approached in order to obtain it (successful in three occasions [14], [17], [20]).

### Quality assessment

Assessment of methodological quality was done by means of a tool based on the Delphi Consensus [21] that we had previously developed [22]. Because of the subjective nature of quality assessments like this, we decided *a priori* to not use the obtained quality scores for excluding or weighing studies in the meta-analysis. We however examined the association between methodological quality and effect size.

The extent of heterogeneity in results between studies was assessed by using the Cochran's Q statistic (with P<0.1 considered to represent statistically significant heterogeneity) and the I^2^-statistic (with 25% considered to be low-level heterogeneity, 25%–50% moderate-level heterogeneity, and >50% high-level heterogeneity) [23], [24].

Publication bias was evaluated by means of visual inspection of funnel plots (constructed by plotting inverse standard errors (SE) against the respective SBP and DBP effects for each trial) and Egger's regression test [25].

### Statistical analysis

For parallel studies, the change in BP was calculated as the change from baseline in the intervention group subtracted from the change from baseline in the control group. For cross-over studies, the change in BP was calculated as the difference between BP at the end of intervention period and BP at the end of the control period. The study of Grassi et al. [13] measured both office and 24-hour ambulatory BP in one control and four treatment periods (each with an increasing dose of tea). To ensure that the weight of this small study was not disproportionately large, we averaged the effects of the four treatments into a single value. The office and ambulatory BP outcomes of this study [13] were entered separately into the analysis, but we halved the weights in these arms so that the office and ambulatory BP outcome each contributed 50% of the overall weight of the study.

The pooled effect and corresponding 95% CIs were calculated by means of fixed and random effects models [26], [27]. Since a random effects model with studies weighted by the inverse of their variance (1/SE^2^) results in a more conservative estimate of statistical significance, this served as the primary model. If SE's of the change in BP were not reported, they were derived from P-values or calculated according to the equations by Follmann et al. [28] assuming a within-subject correlation coefficient of 0.68 [19].

To test whether a particular study was exerting excessive influence on the results, we conducted influence analysis by systematically excluding each study one-by-one and then reanalyzing the remaining data. We also explored the potential effects of pre-defined covariates on the overall outcome by calculating pooled BP effects for different subgroups of dichotomous covariates (only if subgroups consisted of >4 study arms), and by performing weighted meta-regression [29], [30] for continuous covariates. The pre-defined covariates were mean baseline BP (using BP of the control group at the end of the trial period to avoid regression to the mean), dose of tea flavonoids, type of tea (leaf tea or standardized powder), type of placebo (caffeine controlled, yes or no), duration and quality. Calculations were done using the PROC MIXED model of the SAS analytical system (SAS v9.2, SAS Institute, Cary, NC, USA).

## Results

**Figure 1:**
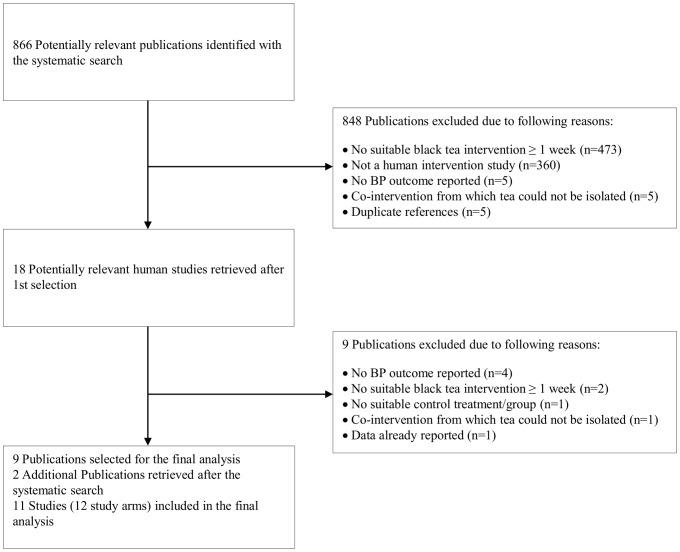
Flow diagram of the study selection procedure.

The systematic searches yielded a total 866 potentially relevant publications (Figure 1). After exclusion of irrelevant publications and studies that did not meet the pre-defined inclusion criteria, 9 studies remained. Two additional eligible studies were published after the conduct of the systematic search [14], [18], yielding a total of 11 studies (12 study arms) that were included in our meta-analysis. Trial characteristics are summarized in **Table 1**.

**Table 1:** Characteristics of 11 controlled intervention studies on tea and blood pressure included in the meta-analysis.

Author	Population 1	Study design ^2^	Study duration (weeks)	Size (n)	Mean age (y)	Mean BMI (kg/m^2^)	Males (%)	BP medication	Intervention product	Dose (ml) ^3^	Dose (mg flavonoids)^3^	Control ^4^	Baseline SBP (mmHg)	Baseline DBP (mmHg)	Quality
Bingham et al. 1997	Healthy	R, S, C	4	65	40.7	24.2	47.7	No	Leaf tea	1800	1494.0	Water (CC)	120.1	75.1	6
Duffy et al. 2001	CAD	R, S, C	4	50	55.0	29.7	78.0	Yes	Extract powder	900	873.0	Water	137.0	77.0	6
Grassi et al. 2012	Hypertensive	R, D, C	1	19	51.3	26.8	26.3	No	Extract powder	400	240.2	Placebo tea (CC)	145.6	92.0	7
Grassi et al. 2009 (office)	Healthy	R, D, C	1	19	32.9	23.9	100.0	No	Extract powder	400	375.0	Placebo tea (CC)	128.8	80.8	7
Grassi et al. 2009 (ABPM)	Healthy	R, D, C	1	19	32.9	23.9	100.0	No	Extract powder	400	375.0	Placebo tea (CC)	121.2	74.4	7
Hodgson et al. 2012	Healthy	R, D, P	26	95	56.6	25.1	34.7	No	Extract powder	600	360.4	Placebo tea (CC)	122.4	73.1	7
Hodgson et al. 2003	Healthy	R, S, C	4	22	59.0	27.0	72.7	No	Leaf tea	1250	1037.5	Water	121.0	72.0	5
Hodgson et al. 2002	MHC	R, S, P	4	21	59.1	27.5	76.2	No	Leaf tea	1250	1037.5	Water	123.0	73.0	6
Hodgson et al. 1999	High-normal SBP	R, S, C	1	13	59.8	27.0	76.9	No	Leaf tea	1000	830.0	Water (CC)	135.5	77.5	6
Mukamal et al 2007	High CVD risk	R, S, P	26	28	65.8	29.1	35.7	Yes	Extract powder	600	318.0	Water	138.4	-	5
Rakic et al 1996	Hypertensive	R, S, P	2	26	73.0	27.4	42.3	Yes	Leaf tea	1250	1037.5	Water	133.2	75.1	4
Schreuder et al. 2013	Healthy	R, S, C	1	20	54.0	25.1	40.0	No	Leaf tea	600	498.0	Water	132.0	78.3	6

1 ABPM, 24-hour ambulatory blood pressure monitoring; CAD, coronary artery disease; MHC, mildly hypercholesterolemic; SBP, systolic blood pressure; CVD, cardiovascular disease; ^2^ R, randomized; S, single blind; C, crossover trial; D, double blind; P, parallel trial; ^3^ Where dose in ml tea or mg flavonoids was not reported, it was calculated according to Astill et al. [49]; ^4^ CC, placebo matched for caffeine content of the tea intervention.

A total of 378 subjects participated in the 11 studies. The number of subjects per study ranged from 13 to 95. Mean age ranged from 33 to 73 years and mean BMI from 24 to 30 kg/m^2^. One study included only men [13]; in the remaining studies, the percentage of male subjects ranged from 26% to 78%. Five of the 11 studies included healthy normotensive populations. The other studies included coronary artery disease patients [31], hypertensive subjects [14], [20], subjects at high cardiovascular risk [17] and subjects with mildly elevated blood pressure [32] and cholesterol [33]. Mean baseline SBP and DBP ranged from 120 to 150 mmHg and from 72 to 92 mmHg, respectively.

Seven studies used a cross-over design and 4 studies a parallel design. Study duration ranged from 1 week to 6 months. The dose of tea consumption in the intervention arms ranged from 400 to 1800 mL per day, providing an estimated 240 to 1500 mg of flavonoids per day. Six of the 11 studies provided intervention products in the form of leaf tea that subjects had to infuse in a defined amount of hot water (200 to 300 mL) for a defined time period (1 to 2 min). In the remaining 5 studies, tea was prepared by dissolving standardized doses of tea extract powder in hot water. In three studies [13]–[15], the control treatment was matched for appearance, taste and caffeine content of the tea interventions, whereas in two studies [32], [34] this was done only for caffeine content. In the remaining six studies [17], [18], [20], [31], [33], [35], the control treatment was plain hot water.

**Figure 2:**
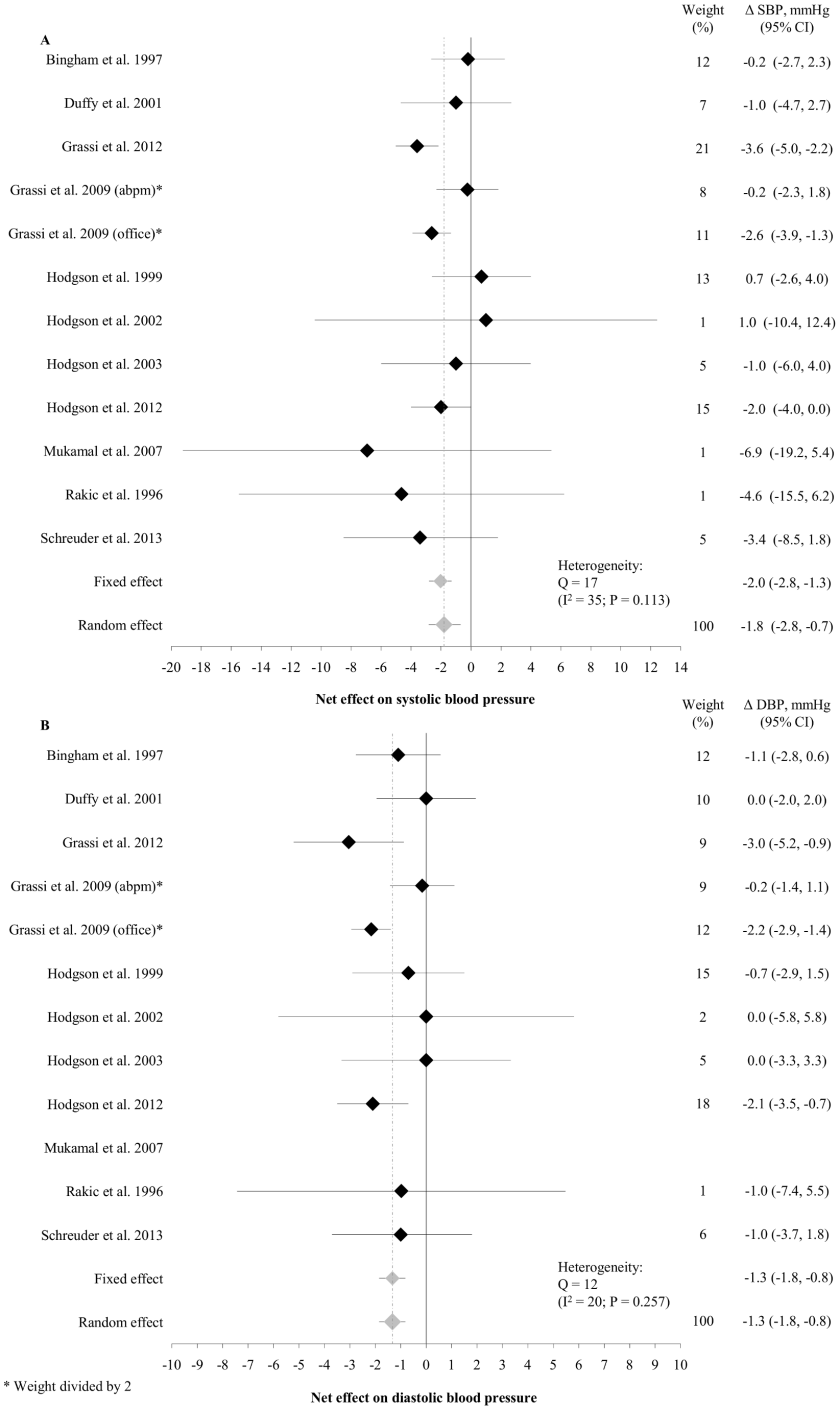
Net changes in SBP (A) and DBP (B) in randomized studies of black tea consumption. Horizontal lines represent 95% confidence intervals. Grey diamonds represent the pooled estimates using fixed and random effects models respectively. Grassi et al. 2009 reported both office and ambulatory BP – both sets of data were included in the analyses, but to keep the weight of the study proportionate, the weight of each arm was halved.

Pooled SBP effects showed moderate heterogeneity (P = 0.11, I^2^ = 35%) between studies, whilst there was no evidence for heterogeneity in DBP effects (P = 0.26, I^2^ = 20%). In the pooled analyses using random effects models, tea intake reduced SBP with 1.8 mmHg (95% CI: −2.8, −0.7; P = 0.0013) and DBP with 1.3 mmHg (95% CI: −1.8, −0.8; P<0.0001). The overall estimates of BP effects were similar when using fixed effects models (−2.0 and −1.3 mmHg for SBP and DBP, respectively) (**Figure 2 A & B**).

In covariate analyses (**Table 2**), studies using tea extract powders found larger reductions in SBP than studies using leaf tea. Baseline SBP and DBP had a significant impact on the effect of tea ingestion on BP, with a ∼1 mmHg larger reduction for every 10 mmHg higher baseline SBP and DBP respectively. Interestingly, each 1-point increase in the perceived quality score of the studies was associated with a 1 mmHg larger reduction in the DBP effect estimate. The covariate analysis found no substantial influence of amount of tea flavonoids, controlling for caffeine content, or the duration of the intervention.

**Table 2:** Covariate analyses - effect of tea on blood pressure within and between different subgroups.

Dichotomous covariates	Change in SBP					Change in DBP				
Subgroup	No. of study arms	Effect	95% CI	P within groups	P between groups	No. of study arms	Effect	95% CI	P within groups	P between groups
Type of intervention										
Powder	6	−2.6	(−3.5; −1.7)	<0.0001*	0.01*	5	−1.7	(−2.3; −1.0)	0.068	0.095
Leaf tea	6	−0.3	(−1.8; 1.2)	0.682		6	−0.8	(−1.6; 0.1)	<0.0001*	
Caffeinated placebo										
Yes	6	−1.7	(−2.9; −0.5)	0.005*	0.886	6	−1.5	(−1.6; 1.0)	0.649	0.09
No	6	−1.9	(−4.4; 0.6)	0.131		5	−0.3	(−2.1; −1.0)	<0.0001*	
Continuous covariates		SBP					DBP			
		β		P			β		P	
Baseline SBP/DBP (per 10 mmHg)		−0.9		0.01*			−1.1		0.04	
Dose (per 100 mg flavonoids)		0.3		0.004*			0.1		0.06	
Duration (per 1 week)		−0.01		0.820			−0.03		0.34	
Quality score (per unit)		−1.3		0.08			−1.0		0.02*	

**Figure 3:**
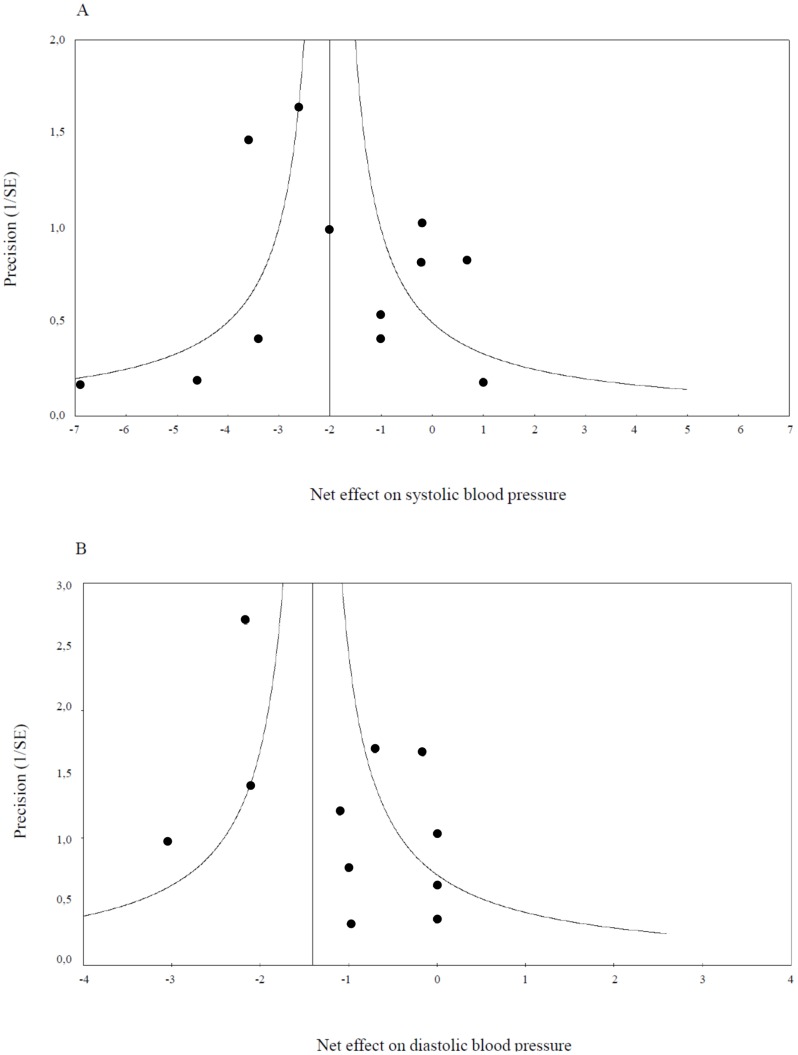
Funnel plots of trials included in the meta-analysis for (A) systolic blood pressure and (B) diastolic blood pressure.

Omitting each study from the analysis to check for disproportionate impact of a single study did not materially affect results for SBP or DBP. Pooled estimates ranged from −1.3 to −2.2 mmHg and −1.2.to −1.5 mmHg for SBP and DBP respectively and remained statistically significant in all cases. Visual inspection of Funnel plots did not indicate the presence of publication bias and this was supported by the outcome of Egger's linear regression test (intercept: 0.56; P = 0.45 for SBP and intercept: 0.76; P = 0.37 for DBP) (**Figure 3 A and B**).

## Discussion

This meta-analysis, summarizing evidence from 11 randomized controlled intervention studies including 378 subjects, found that daily consumption of black tea for ≥ 1 week is associated with a statistically significant reduction in SBP and DBP of 2 and 1 mmHg, respectively. Although these reductions in BP are modest from an individual's perspective, such reductions are relevant on a population-wide scale and may translate to substantial reductions in CVD risk [3], [4]. Moreover, we found that the effect of tea ingestion was more pronounced in those with a higher *a priori* baseline BP. In fact, data from the Prospective Studies Collaboration indicate that a 2 mmHg lower SBP is associated with a 10% lower stroke mortality and about 7% lower mortality from ischemic heart disease or other vascular causes in middle age subjects [3]. Therefore, our findings provide a possible explanation for the reductions in risk for stroke observed in epidemiological studies on tea consumption [36]–[38].

Our rationale for conducting this meta-analysis was the recent publication of a number of high-quality randomized controlled trials, which could appreciably change the outcomes from previous meta-analyses and the general consensus from those analyses that tea may not impact BP. Three of the newly included studies reported statistically significant effects [13]–[15]. These studies had well-standardized interventions, robust double-blind designs with repeated BP measurements and low variances causing them to contribute a large amount of weight to the analysis. This explains our observation of a relation between the perceived quality score of the studies and the reported effect size of tea on BP. More specifically, studies with higher quality scores generally reported lower BP variances which mean higher statistical power to detect statistically significant reductions in BP after tea intake. It is thus important for future studies on tea and BP to achieve the highest possible level of quality.

A biological mechanism that could, at least in part, explain the BP lowering effect of black tea relates to the influence of black tea flavonoids on endothelial function. Previous studies found that endothelial function shares a complex relationship with hypertension and it has been suggested as an early marker for BP changes [39]. Human intervention studies consistently indicate that black tea consumption improves endothelial function as measured by brachial artery flow mediated dilation (FMD) [22], most likely through improved bioavailability of nitric oxide (NO) [40], [41]. Accordingly, the decrease in BP after daily tea ingestion may be, at least partly, mediated through an improvement in endothelial function, which may contribute to a lowering in peripheral vascular resistance.

The magnitude of the effect of tea ingestion on BP may be dependent on baseline BP. We found that each 10 mmHg higher baseline SBP was associated with a 1 mmHg larger SBP effect size and a similar association was seen for DBP. The dependence of treatment effect size on baseline BP has been reported previously [42]–[45]. This would imply that individuals with a higher resting BP and therefore higher risk for CVD would benefit most from measures to reduce BP, including tea consumption. This observation might have been due to the regression to the mean effect since it is known to be a serious confounder in blood pressure studies. Our statistical approach should have avoided this however and the control and intervention arms of the studies included in our analyses were well matched for baseline BP levels.

Our study found no evidence for a substantial influence of the duration of the intervention or the amount of daily flavonoid intake on the BP effect. Kay et al. meta-analyzed data from 77 studies of various flavonoid sources (tea, but also chocolate, red wine, berries or grapes and flavonoid extracts) to relate BP and FMD effects with flavonoid contents. In agreement with our study, no dose-response relation could be detected between flavonoid intake and BP [7]. In the American Cancer Society's CPS-II Nutrition Cohort, McCullough et al. found nonlinear associations between flavonoid intake and fatal CVD, with lower risk already apparent in the second intake quintile [46]. Combined with our current meta-analysis, this suggests that beneficial effects of flavonoid-rich foods like black tea on BP and CVD risk could be already attained at moderate intake levels.

Several limitations of this meta-analysis should be noted. Firstly, the majority of the included studies had relatively small sample sizes, potentially leading to publication bias and overestimation of treatment effects, because smaller trials might be methodologically less robust and are prone to report larger effect sizes [47], [48]. However, we could not detect evidence of systematic publication bias in our analysis although such bias can never be fully ruled out. Secondly, only three of the included studies were double-blinded. It is well possible that detection bias has occurred in the other studies where the subjects and, in some cases, the investigators were not blinded as to the treatments. Interestingly, in our analysis a larger effect size of tea ingestion on BP was related with a high quality rating. This indicates that the BP lowering effect of tea estimated here cannot be simply explained by the inclusion of the un-blinded, lower quality studies. Finally, the outcomes of the covariate analyses on factors influencing the BP effect of tea should be interpreted with caution because our meta-analysis included a relatively small number of studies, and was therefore not suited to reliably estimate the impact of potential covariates.

In conclusion, this review provides an up-to-date overview of controlled human intervention studies on the effects of black tea consumption on BP. Our analysis indicates that daily consumption of 4 to 5 cups of black tea can reduce BP by 1–2 mmHg. Although this effect is modest, it may be of importance for cardiovascular health at the population level because of the widespread consumption of black tea and the high prevalence of hypertension and consequent risk of CVD.

## Supporting Information

Checklist S1 **PRISMA Checklist.**
(DOCX)
